# Optimum mating designs for exploiting dominance in genomic selection schemes for aquaculture species

**DOI:** 10.1186/s12711-021-00610-9

**Published:** 2021-02-10

**Authors:** Jesús Fernández, Beatriz Villanueva, Miguel Angel Toro

**Affiliations:** 1 Departamento de Mejora Genética Animal, INIA, Madrid, Spain; 2 Departamento de Producción Agraria, ETSI Agronómica, Alimentaria y de Biosistemas, UPM, Madrid, Spain

## Abstract

**Background:**

In commercial fish, dominance effects could be exploited by predicting production abilities of the offspring that would be generated by different mating pairs and choosing those pairs that maximise the average offspring phenotype. Consequently, matings would be performed to reduce inbreeding depression. This can be achieved by applying mate selection (MS) that combines selection and mating decisions in a single step. An alternative strategy to MS would be to apply minimum coancestry mating (MCM) after selection based on estimated breeding values. The objective of this study was to evaluate, by computer simulations, the potential benefits that can be obtained by implementing MS or MCM based on genomic data for exploiting dominance effects when creating commercial fish populations that are derived from a breeding nucleus.

**Methods:**

The selected trait was determined by a variable number of loci with additive and dominance effects. The population consisted of 50 full-sib families with 30 offspring each. Males and females with the highest estimated genomic breeding values were selected in the nucleus and paired using the MCM strategy. Both MCM and MS were used to create the commercial population.

**Results:**

For a moderate number of SNPs, equal or even higher mean phenotypic values are obtained by selecting on genomic breeding values and then applying MCM than by using MS when the trait exhibited substantial inbreeding depression. This could be because MCM leads to high levels of heterozygosity across the whole genome, even for loci affecting the trait that are in linkage equilibrium with the SNPs. In contrast, MS specifically promotes heterozygosity for SNPs for which a dominance effect has been detected.

**Conclusions:**

In most scenarios, for the management of aquaculture breeding programs it seems advisable to follow the MCM strategy when creating the commercial population, especially for traits with large inbreeding depression. Moreover, MCM has the appealing property of reducing inbreeding levels, with a corresponding reduction in inbreeding depression for traits beyond those included in the selection objective.

## Background

Non-additive genetic effects are usually ignored in animal breeding programmes for several reasons [1]. Perhaps the main reason is the scarcity of suitable data for estimating these effects, in particular the scarcity of informative pedigrees. However, the structure of the populations used in aquaculture breeding programs, with large full-sib families, may facilitate estimation of dominance effects. In fact, significant dominance effects have been detected for traits of commercial interest in aquaculture, such as growth traits [2–5] and filet yield [5].

The availability of genomic information may make the estimation of dominance effects easier, and several authors have included dominance effects in genomic evaluation models [6–16]. However, even when using genomic data, the accuracy of estimates of dominance effects still depends on the number of individuals sampled and the structure of the population (in particular, the presence or not of large full-sib families) [17].

In aquaculture programs, the breeding population (i.e. the nucleus in which selection is performed) is usually kept separate from the commercial population that is composed of fish that are destined for market. The reason for this dual structure is that the production levels of the nucleus may be not able to provide the number of fish required to cope with the demand. Therefore, a population that is separate from the nucleus must be created at each generation.

The main objective in the breeding nucleus is to identify and select the fish with the highest breeding values to produce the next generation. The interest in estimating dominance effects in the nucleus would be to increase the accuracy of estimates of breeding values [6]. However, the main objective when creating a commercial population from the nucleus is to maximise the phenotypic performance of the fish destined for market. Thus, for commercial fish, dominance effects could be explicitly exploited by predicting production abilities of the offspring that would be generated by different mating pairs and choosing those pairs that maximise the average offspring phenotype. Consequently, matings would be performed to reduce inbreeding depression (an important consequence of the dominance) [6, 14–16, 18]. This objective can be achieved by applying an optimisation method such as mate selection (MS) that combines selection and mating decisions in a single step [19–23].

Minimum coancestry mating (MCM) following selection has also proved to be an efficient strategy for controlling inbreeding in selection programmes [24, 25]. Effective control of inbreeding would reduce the risk of inbreeding depression in the commercial population. Thus, an alternative strategy to MS for creating commercial populations would be to apply MCM after selection based on estimated breeding values.

As suggested in the literature, estimation of breeding values and dominance deviations to predict the expected offspring genotype required for MS could benefit from the use of the high-density single nucleotide polymorphism (SNP) genotyping tools that are currently available [6, 17]. With sufficient markers, the genomic relationship matrices for both additive and dominance effects are expected to be more accurate than pedigree-based matrices. Similarly, the coancestry coefficients required to perform MCM (where the average pairwise coancestry coefficient in the selected group is minimized) are expected to have a higher accuracy when computed from genomic data than from pedigree data [26, 27].

The objective of this study was to evaluate the potential benefits that can be obtained by implementing MS or MCM based on genomic data for exploiting dominance effects when creating commercial fish populations that are derived from a breeding nucleus. Stochastic simulation was used to achieve these objectives.

## Methods

### Genome structure

Diploid individuals with a genome of 15 M and comprising 24 chromosomes of equal size were simulated. Such a genome is similar to that of some of the most common aquaculture species (e.g., sea bream, sea bass and turbot). In total, 200,000 biallelic loci were homogenously distributed across chromosomes and evenly spaced on each chromosome. One hundred to 200,000 loci, with equal numbers per chromosomes, were used to evaluate the selection candidates and to decide how the selected fish were mated. These loci are referred to as SNPs and were simulated at even distances along the chromosomes. Genotypes for non-SNP loci were used to monitor the effect of the strategies evaluated on genetic diversity (expected heterozygosity and genomic inbreeding).

### Generation of selection candidates

The creation of fish in the base population from which the breeding program started followed a two-step process. First, a large population in mutation-drift equilibrium was generated, and second, individuals were sampled from this population to constitute the base population.

#### Equilibrium population

In order to create linkage disequilibrium (LD) between loci, a large population ($N$ = 1000, 50% males and 50% females) with random mating was simulated for 3000 discrete generations. For each generation, sires and dams of the new offspring were randomly sampled with replacement. Population size was kept constant across generations. To generate individuals for the starting population, genotypes for each locus were sampled at random, with initial allelic frequencies equal to 0.5 for all loci and, thus, the initial population was in Hardy–Weinberg and linkage equilibrium. Across generations, mutation was allowed to occur throughout the genome, with a mutation rate per locus and generation of $\mu$ = 2.5 $\times$ 10^–3^ for both SNPs and non-SNPs. The number of new mutations simulated in each generation was sampled from a Poisson distribution with a mean equal to $2N{n}_{c}\mu {n}_{l}$, where ${n}_{c}$ is the number of chromosomes and ${n}_{l}$ is the total number of loci per chromosome. Mutations were then randomly distributed across individuals, chromosomes and loci, switching allele 0 to allele 1 and vice versa. When generating a gamete, the total number of crossovers was drawn from a Poisson distribution with a mean equal to 15. Crossovers were randomly distributed across the genome without interference. At the end of the 3000 generations, the expected heterozygosity (${H}_{e}$) of the population had stabilized around an equilibrium value (${H}_{e}$ = 0.48) and the mean LD between adjacent loci, measured by ${r}^{2}$, was 0.03. At equilibrium, all loci were still segregating.

#### Base population

The second step of the process consisted of randomly sampling 100 individuals from the population at equilibrium. These fish constituted the founders of the breeding program. At this step, a quantitative trait with an initial heritability ${h}_{(0)}^{2}$ of 0.4 was defined. Phenotypic mean and variance were 0 and 1, respectively. When dominance was simulated, the proportion of phenotypic variance explained by dominance effects (${d}_{(0)}^{2})$ was set to 0.2. In most of the scenarios, the trait, which was measured on both sexes, was controlled by 1000 loci (thereafter called quantitative trait loci, QTL) with no epistatic interactions between them. Scenarios in which the trait was controlled by 10 or 100 QTL were also simulated. In all cases, QTL were chosen at random from the simulated SNPs and non-SNPs. Two types of scenarios were simulated: (i) the effects of all QTL were equal (EQU); and (ii) the effects varied across QTL (VAR). In the EQU scenarios, two types of gene action were considered, which differed in the values given to the additive effect (${a}^{*}$, the value of the homozygous 11 genotype), and the dominance effect (${d}^{*}$, the value of the heterozygous genotype):

- Additive (ADD_EQU): ${a}^{*}=1$ and ${d}^{*}=0$ for all QTL,
- Dominant (DOM_EQU_ $\mathrm{x}$): ${a}^{*}={d}^{*}=1$ for all QTL,

where $\mathrm{x}$ represents the number of QTL that control the trait: 10 (DOM_EQU_10), 100 (DOM_EQU_100) or 1000 (DOM_EQU_1000). Thus, three levels of inbreeding depression were simulated (the larger the number of QTL, the higher the inbreeding depression).

In the VAR scenarios, three gene actions were considered:

- Additive (ADD_VAR): the additive effect of QTL $i$ (${a}_{i}^{*}$) was sampled from a normal distribution with mean 0 and variance 1; no dominance effects were simulated.
- Dominant (DOM_VAR): both additive and dominance effects (${a}_{i}^{*}$ and ${d}_{i}^{*}$) were sampled independently from normal distributions with mean 0 and variance 1. Note that ${d}_{i}^{*}$ may be positive, negative or zero, and thus the QTL may be (over-, under-) dominant, recessive or additive. In this situation of no directional dominance, no inbreeding depression is expected.
- Directional dominance (DIR_VAR): both additive and dominance effects (${a}_{i}^{*}$ and ${d}_{i}^{*}$) were sampled independently from normal distributions with mean 0 and variance 1 but then the absolute value was taken for each ${d}_{i}^{*}$, resulting in directional dominance and inbreeding depression.

In all scenarios, the final additive effect for QTL $i$ (${a}_{i}$) was obtained by multiplying ${a}_{i}^{*}$ by the factor $\sqrt{\left\{{V}_{A(0)}/\left[2p(1-p){n}_{QTL}\right]\right\}}$, where ${V}_{A(0)}$ is the initial additive variance (i.e. ${h}_{(0)}^{2}{V}_{P(0)}=0.4)$, $p$ is the average frequency across QTL in the base population, and ${n}_{QTL}$ is the number of QTL (i.e. 1000, 100 or 10). Note that this results in the expected additive variance summed over all loci to equal ${V}_{A(0)}$, assuming that covariances between loci generated by LD are negligible and given that the allele frequencies at $t$ = 0 are close to 0.5 and, therefore, the allele substitution effect ${\alpha }_{i}\approx {a}_{i}$. Similarly, the final dominance effects (${d}_{i}$) were obtained by multiplying ${d}_{i}^{*}$ by $\left\{1/\left[2p(1-p)\right]\right\}\sqrt{\left({V}_{D(0)}/{n}_{QTL}\right)}$, with ${V}_{D(0)}=0.2$. Additive and dominance effects were constant across all generations of selection.

The phenotypic value for an individual $j$ was obtained as the sum across QTL $i$ of ${a}_{i}$, ${d}_{i}$ or ${-a}_{i}$ for homozygotes 11, heterozygotes 10, and homozygotes 00, respectively, and an individual environmental deviation that was sampled from a normal distribution with mean 0 and variance ${V}_{E}=1-\left({V}_{A(0)}+{V}_{D(0)}\right)$. The environmental variance (${V}_{E}$) was initially calculated for each replicate in order to ensure that each replicate started with the same proportion of genetic variance, and was kept constant across generations.

### Breeding program

The breeding program included a breeding nucleus (where selection was performed) and a commercial population (composed by individuals that are slaughtered) that was obtained from the nucleus at each generation. The selection for four discrete generations was simulated. To create generation $t$ = 0 (i.e. the base population), founders were mated at random to form 50 full-sib families, and 30 offspring (half of each sex) were obtained from each mated pair. Consequently, the number of selection candidates in this first generation (and in subsequent generations) was 1500. Genotypes and phenotypes were available for all candidates.

### Genetic evaluation

Genomic evaluations were performed using phenotypes and genotypes of selection candidates in the nucleus at a given generation and of individuals of previous generations. The following genomic BLUP_SNP model using the software GS3 [28] was assumed to estimate breeding values and dominance deviations:

1 $${\mathbf{y}}=\mathbf{1}\mu +{\mathbf{Zu}}+{\mathbf{Wv}}+b{\mathbf{F}}+{\mathbf{e}}$$

where $\mathbf{y}$ is the vector of phenotypes, $\mathbf{1}$ is a vector of 1s, $\mu$ is the population mean, $\mathbf{u}$ and $\mathbf{v}$ are the vectors of additive and dominance SNP effects, respectively, $\mathbf{F}$ is a vector with mean SNP heterozygosity of each individual, $b$ the regression coefficient of phenotypes on heterozygosity, $\mathbf{e}$ is the vector of random residuals, and $\mathbf{Z}$ and $\mathbf{W}$ are incidence matrices constructed from the SNP genotypes for each individual. The element of the $i$ th row and $j$ th column in $\mathbf{Z}$ was 2, 0 or 1, if the $i$ th fish was respectively homozygous 11, heterozygous 10, or homozygous 00 for SNP $j$. The corresponding element of $\mathbf{W}$ was 0 if the fish was homozygous and 1 if the fish was heterozygous. It was assumed that SNP effects were uncorrelated and that $\mathrm{V}\left(\mathbf{u}\right)=\mathbf{I}{\sigma }_{\mathrm{u}}^{2}$ and $\mathrm{V}\left(\mathbf{v}\right)=\mathbf{I}{\sigma }_{\mathrm{v}}^{2}$, where $\mathbf{I}$ is the identity matrix and ${\sigma }_{\mathrm{u}}^{2}$ and ${\sigma }_{\mathrm{v}}^{2}$ are the variances of the additive and dominance SNP effects. Variance ${\sigma }_{\mathrm{u}}^{2}$ was calculated by dividing the simulated total additive variance by $2\sum \left[{p}_{i}\left(1-{p}_{i}\right)\right]$, where ${p}_{i}$ is the frequency, at $t$ = 0, of allele 1 of SNP $i$ in the group of individuals evaluated [29]. For ${\sigma }_{\mathrm{v}}^{2}$, the weighting term in the denominator was $4\sum \left[{p}_{i}^{2} {\left(1-{p}_{i}\right)}^{2}\right]$. Both ${\sigma }_{\mathrm{u}}^{2}$ and ${\sigma }_{\mathrm{v}}^{2}$ were kept constant across generations. The model included the effect of observed heterozygosity of the candidates as a covariate to account for inbreeding depression [14, 15].

The estimated breeding value (EBV) for each fish was calculated from the estimated additive SNP effects (${\widehat{a}}_{i}$), and the dominance effects (${\widehat{d}}_{i}$) by summing estimated additive genetic values across the SNPs. For SNP $i$, estimated additive genetic values were equal to $2\left(1-{p}_{i}\right){\widehat{\alpha }}_{i}$, $\left(1-2{p}_{i}\right){\widehat{\alpha }}_{i}$, or $-{p}_{i}{\widehat{\alpha }}_{i}$ for homozygotes 11, heterozygotes 10, and homozygotes 00, respectively, where ${\widehat{\alpha }}_{i}={\widehat{a}}_{i}+\left(1-2{p}_{i}\right){\widehat{d}}_{i}$. Similarly, the estimated dominance deviation for each fish was calculated from the estimated dominance SNP effects (${\widehat{d}}_{i}$) by summing estimates of dominance deviations across the SNPs, which were equal to $-2{\left(1-{p}_{i}\right)}^{2}{\widehat{d}}_{i}$, $2{p}_{i}\left(1-{p}_{i}\right){\widehat{d}}_{i}$, or $-2{p}_{i}^{2}{\widehat{d}}_{i}$ for homozygotes 11, heterozygotes 10, and homozygotes 00, respectively. True breeding values (TBV) and dominance effects were calculated in the same way but using the effects and frequencies of QTL.

### Selection and mating in the nucleus

The 50 males and 50 females with the highest EBV were selected to be the parents of the next generation in the nucleus. Each selected fish was involved in one mating only. Matings followed a minimum coancestry mating (MCM) design to form 50 full-sib families of 30, for a total of 1500 offspring, which constituted the selection candidates for the next generation. Optimization in the MCM scheme was performed by a simulated annealing algorithm [30], using the molecular coancestry matrix based on the proportion of SNP alleles shared by two individuals [31, 32]. Note that only the 50 selected males and 50 selected females were involved in the optimization.

#### Selection and mating to produce the commercial population

Two strategies were used to create the fish that constituted the commercial population. In the first strategy, commercial fish were generated in the same way as the selection candidates in the nucleus; i.e., selection based on EBV and selected fish mated using the MCM scheme. In the second strategy, commercial fish were generated in a single step process known as ‘mate selection’ (MS), taking advantage of dominance effects. In this strategy, all selection candidates (750 males and 750 females) enter the optimization.

To implement MS, first, a matrix with the expected phenotypic value of the offspring of each possible mating pair was created. This value depends on the SNP genotypes of the parents, which determine the probability of transmitting allele 1 or 0. Homozygous parents 11 always transmit allele 1, homozygous parents 00 never transmit allele 1, and heterozygous parents transmit allele 1 to half of their offspring. The proportion of offspring with each possible genotype ${\mathrm{P}}_{11}$, ${\mathrm{P}}_{10}$ and ${\mathrm{P}}_{00}$ for genotypes 11, 10 and 00, respectively, was calculated by multiplying the probabilities of transmitting each allele (assuming no deviations from Mendelian inheritance) for each mating pair. Then, the expected phenotype of the offspring of a potential mating pair was estimated as the sum across each SNP $i$ of ${\widehat{a}}_{i}{\mathrm{P}}_{11}+{\widehat{d}}_{i}{\mathrm{P}}_{10}-{\widehat{a}}_{i}{\mathrm{P}}_{00}$. Finally, the expected heterozygosity in the offspring (calculated as 1 minus the molecular coancestry between the parents) multiplied by the regression coefficient estimated in the evaluation step was added to the matrix of expected phenotypes of the offspring, such that mating pairs that result in lower levels of SNP-based inbreeding were given higher priority.

In order to be comparable with the MCM strategy, the combination of 50 mating pairs that yielded the highest average expected phenotypes in the offspring (from all 750 $\times$ 750 possible matings) were selected and mated, and 30 offspring were generated from each mating. The optimization was performed using a Hungarian algorithm [33].

Finally, in order to have a benchmark scenario to better understand the performance of both the MCM and the MS strategy, all simulations were also run using the true (simulated) additive and dominance values for the QTL, both in the management of the nucleus and the creation of the commercial population. These scenarios will be referred to as QTL scenarios in contrast with SNP scenarios in which marker information is used.

Gametes from each of the parents to create offspring were generated following the same procedure as explained before for the equilibrium population, except that no mutation was allowed during the selection period.

### Parameters compared

The main comparisons were based on the phenotypic mean obtained in the commercial population each generation under the MS and MCM strategies. The average genealogical inbreeding coefficient and the observed homozygosity at SNPs, non-SNPs, and QTL obtained under the different strategies were also compared. The accuracies of the estimates of the breeding and dominance values were calculated as the correlation between the true (using the genotypes and the frequencies at the QTL) and the estimated (using the estimated effects and the actual allelic frequencies at the SNPs) values. Results presented are the averages of 100 replicates.

## Results

The average phenotypic performance of the commercial population under both MCM and MS strategies for each simulated scenario is in Table 1 for generations 1 and 4. The results of the simulations in which additive gene action was assumed (whether ADD_EQU or ADD_VAR) followed very similar patterns and, thus, only those for the ADD_VAR scenario are shown.

**Table 1:** Mean phenotype in the commercial population at generations ($t$) 1 and 4 when using MCM or MS based on the genotypes of a variable number of SNPs (*n*_*SNP*_) for different types of gene action

*n*_*SNP*_	ADD_VAR		DOM_VAR		DOM_EQU_10		DOM_EQU_100		DIR_VAR		DOM_EQU_1000	
	MCM	MS	MCM	MS	MCM	MS	MCM	MS	MCM	MS	MCM	MS
*t* = 1												
100	0.60	0.60	0.57	0.60	0.50	0.51	0.55	0.50	0.82	0.52	0.61	0.39
1000	0.78	0.77	0.74	0.80	0.60	0.55	0.71	0.45	0.83	0.23	0.86	0.12
10,000	0.81	0.81	0.79	0.88	0.62	0.48	0.76	0.08	0.92	− 0.73	0.94	− 1.03
100,000	0.81	0.81	0.80	0.90	0.65	0.46	0.78	− 0.05	0.93	− 1.17	0.97	− 1.65
200,000	0.87	0.87	0.84	0.96	0.69	0.53	0.84	− 0.01	0.98	− 1.17	1.02	− 1.63
*t* = 4												
100	1.64	1.64	1.69	1.71	1.06	1.04	1.28	1.17	1.47	1.15	0.98	0.66
1000	2.58	2.57	2.59	2.63	1.31	1.29	2.01	1.90	2.09	1.79	1.96	1.55
10,000	2.80	2.80	2.85	2.92	1.36	1.34	2.23	2.07	2.39	1.92	2.27	1.75
100,000	2.81	2.80	2.88	2.96	1.36	1.35	2.24	2.05	2.42	1.88	2.33	1.68
200,000	2.86	2.85	2.94	3.03	1.38	1.36	2.30	2.10	2.44	1.89	2.39	1.75

Standard errors ranged from 0.02 to 0.13

*MCM* minimum coancestry mating, *MS* mate selection, *ADD_VAR* only additive effects normally distributed, *DOM_VAR* additive and dominance effects normally distributed, *DIR_VAR* as in DOM_VAR but with dominance effects all positive, *DOM_EQU_x* all effects equal with *d* = *a* being × the number of QTL (actually 10, 100 or 1000)

In all MCM scenarios, phenotypic values and accuracies of EBV and dominance deviations (Table 2) increased as the number of SNPs used increased. However, increasing the number of SNPs genotyped above 10,000 led to little additional improvement in accuracy and response, although the 200,000 scenario included the genotypes of all QTL. Except for low SNP densities, the accuracy of EBV remained constant across generations. Please note that estimation of SNP effects was performed for each generation and, thus, linkage between QTL and SNPs was reevaluated at each generation, resulting in this maintenance of accuracy.

**Table 2:** Accuracy of estimates of breeding values (Add) and dominance deviations (Dom) based on the genotypes of a variable number of SNPs (*n*_*SNP*_) at generations ($t$) 1 and 4 for different types of gene action

*n*_*SNP*_	ADD_VAR		DOM_VAR		DOM_EQU_10		DOM_EQU_100		DIR_VAR		DOM_EQU_1000	
	Add	Dom	Add	Dom	Add	Dom	Add	Dom	Add	Dom	Add	Dom
*t* = 1												
100	0.53	–	0.47	0.16	0.47	0.16	0.46	0.16	0.57	0.28	0.42	0.23
1000	0.73	–	0.68	0.32	0.68	0.32	0.67	0.32	0.62	0.37	0.59	0.39
10,000	0.76	–	0.73	0.42	0.72	0.42	0.71	0.43	0.66	0.47	0.63	0.50
100,000	0.77	–	0.73	0.43	0.73	0.43	0.72	0.45	0.66	0.49	0.63	0.51
200,000	0.77	–	0.73	0.43	0.73	0.45	0.72	0.44	0.66	0.49	0.66	0.51
*t* = 4												
100	0.32	–	0.32	0.09	0.26	0.08	0.30	0.09	0.39	0.13	0.30	0.10
1000	0.65	–	0.63	0.27	0.39	0.21	0.57	0.27	0.58	0.30	0.57	0.29
100,00	0.74	–	0.73	0.46	0.45	0.36	0.66	0.46	0.67	0.47	0.66	0.47
100,000	0.75	–	0.74	0.50	0.47	0.40	0.67	0.50	0.69	0.52	0.68	0.51
200,000	0.75	–	0.74	0.51	0.46	0.40	0.68	0.51	0.69	0.51	0.69	0.53

Standard errors were lower than 0.02

*ADD_VAR* only additive effects normally distributed, *DOM_VAR* additive and dominance effects normally distributed, *DIR_VAR* as in DOM_VAR but with dominance effects all positive, *DOM_EQU_x* all effects equal with *d* = *a* being × the number of QTL (actually 10, 100 or 1000)

Both MCM and MS strategies performed equally when gene action was additive (Table 1), which is as expected since the predicted mean phenotype in the offspring with pure additivity depends on selection decisions and not on the mating system. However, differences in the observed molecular homozygosity (Table 3) and genealogical inbreeding were found (see Additional file 1: Table S1). Whereas MCM explicitly avoids the generation of molecular inbreeding at the SNPs (and indirectly at the other loci because markers cover the whole genome) and of genealogical inbreeding, *MS* generates more inbreeding. In fact, for example, at $t$ = 1, the genealogical inbreeding was still zero under MCM but greater than zero under MS.

**Table 3:** Mean observed homozygosity (${H}_{o}$) for the non-SNP loci and the QTL (in italics) in the commercial population when using the MCM or MS strategy based on the genotypes of a variable number of SNPs (*n*_*SNP*_) under different gene actions

*n*_*SNP*_	ADD_VAR		DOM_VAR		DOM_EQU_10		DOM_EQU_100		DIR_VAR		DOM_EQU_1000	
	MCM	MS	MCM	MS	MCM	MS	MCM	MS	MCM	MS	MCM	MS
*t* = 1												
100	0.52	0.57	0.52	0.54	0.52	0.53	0.52	0.54	0.52	0.54	0.52	0.53
	*0.52*	*0.57*	*0.52*	*0.54*	*0.53*	*0.54*	*0.52*	*0.53*	*0.52*	*0.53*	*0.52*	*0.53*
1000	0.52	0.58	0.52	0.56	0.52	0.55	0.52	0.56	0.52	0.55	0.52	0.55
	*0.52*	*0.58*	*0.52*	*0.56*	*0.54*	*0.56*	*0.52*	*0.55*	*0.52*	*0.55*	*0.52*	*0.55*
10,000	0.52	0.58	0.52	0.62	0.52	0.61	0.52	0.61	0.52	0.60	0.52	0.59
	*0.52*	*0.58*	*0.52*	*0.62*	*0.54*	*0.59*	*0.52*	*0.60*	*0.52*	*0.60*	*0.52*	*0.59*
100,000	0.52	0.58	0.52	0.63	0.52	0.63	0.52	0.63	0.52	0.62	0.52	0.62
	*0.52*	*0.58*	*0.52*	*0.63*	*0.54*	*0.60*	*0.52*	*0.61*	*0.52*	*0.62*	*0.52*	*0.61*
200,000	0.52	0.58	0.52	0.63	0.52	0.63	0.52	0.63	0.52	0.62	0.53	0.62
	*0.52*	*0.58*	*0.52*	*0.63*	*0.54*	*0.60*	*0.52*	*0.61*	*0.52*	*0.62*	*0.52*	*0.62*
*t* = 4												
100	0.55	0.57	0.56	0.57	0.55	0.56	0.55	0.56	0.55	0.56	0.55	0.56
	*0.56*	*0.57*	*0.56*	*0.57*	*0.62*	*0.63*	*0.55*	*0.56*	*0.55*	*0.56*	*0.54*	*0.55*
1000	0.55	0.59	0.55	0.57	0.54	0.55	0.54	0.56	0.54	0.56	0.54	0.56
	*0.55*	*0.59*	*0.55*	*0.57*	*0.70*	*0.70*	*0.55*	*0.57*	*0.54*	*0.56*	*0.54*	*0.55*
10,000	0.55	0.61	0.54	0.58	0.54	0.56	0.54	0.57	0.54	0.56	0.54	0.56
	*0.55*	*0.61*	*0.55*	*0.58*	*0.74*	*0.74*	*0.56*	*0.57*	*0.54*	*0.56*	*0.54*	*0.56*
100,000	0.54	0.60	0.54	0.59	0.54	0.57	0.54	0.58	0.54	0.57	0.54	0.57
	*0.55*	*0.60*	*0.55*	*0.59*	*0.74*	*0.74*	*0.56*	*0.58*	*0.54*	*0.56*	*0.54*	*0.56*
200,000	0.55	0.60	0.55	0.59	0.54	0.57	0.54	0.58	0.54	0.57	0.55	0.57
	*0.55*	*0.60*	*0.55*	*0.59*	*0.75*	*0.75*	*0.56*	*0.58*	*0.54*	*0.56*	*0.54*	*0.56*

Standard errors lower than 0.01

*MCM* minimum coancestry mating, *MS* mate selection, *ADD_VAR* only additive effects normally distributed, *DOM_VAR* additive and dominance effects normally distributed, *DIR_VAR* as in DOM_VAR but with dominance effects all positive, *DOM_EQU_x* all effects equal with *d* = *a*, being × the number of QTL (actually 10, 100 or 1000)

**Fig. 1:**
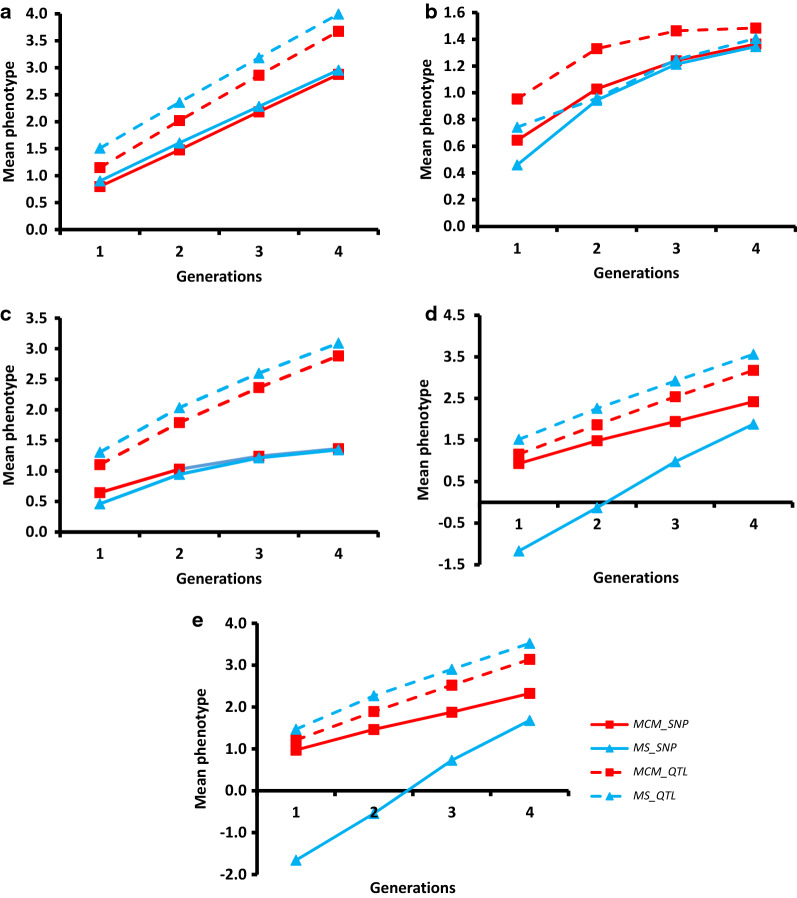
Mean phenotype in the commercial population across generations when using the MCM and MS strategies based on genotypes for 100,000 SNPs (strategies _SNP) or the true additive and dominant effect and the genotypes of the QTL (strategies _QTL). **a** scenario DOM_VAR; **b** scenario DOM_EQU_10; **c** scenario DOM_EQU_100; **d** scenario DIR_VAR; and **e** scenario DOM_EQU_1000

**Fig. 2:**
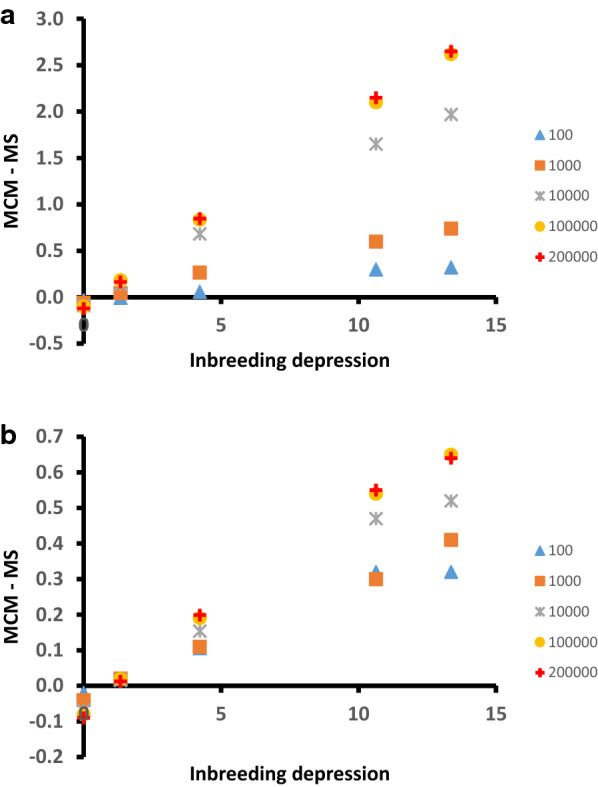
Difference between the mean phenotype when using the MCM and MS strategies with different numbers of SNPs plotted against the inbreeding depression of the simulated trait (in standard deviations per 100% of increase in inbreeding). **a** generation one; and **b** generation four

Although the aim of MS is to take dominance effects into account to obtain the highest possible mean phenotypes in the commercial population, for most scenarios with dominance (DIR_VAR, DOM_EQU_10, DOM_EQU_100 and DOM_EQU_1000), it performed clearly worse than MCM (Table 1 and Fig. 1). Only in the case of no directional dominance (DOM_VAR), did MS outperform MCM. The superiority of MCM increased with increasing levels of inbreeding depression, as shown in Fig. 2, where the difference between the performance of MCM and MS at $t$ = 1 and $t$ = 4 is shown for scenarios with dominance effects.

This suggests that the poor performance of MS is related to the higher levels of inbreeding generated with this strategy. In addition, the difference between the performance of MCM and MS increased with the number of SNPs used (Fig. 2).

The observed homozygosity (${H}_{o}$) was always higher for MS than for MCM, irrespective of the type of loci used for the calculations (Table 3) and (see Additional file 1: Table S1). Under both management strategies, ${H}_{o}$ measured at the SNPs was practically equal to ${H}_{o}$ measured at the non-SNP loci (see Additional file 1: Table S1). The only significant differences were observed for scenarios in which only 100 SNPs were used for genetic evaluation, for which SNP ${H}_{o}$ was lower at $t$ = 1 under MCM and higher at $t$ = 4 under MS. Few differences were observed between ${H}_{o}$ calculated at non-SNP loci and at QTL, except for scenario DOM_EQU_10 (Table 3). For both MS and MCM, ${H}_{o}$ was higher at QTL, especially in the last generation and when a large number of SNPs was used for genetic evaluation.

Evolution of genealogical inbreeding (and molecular homozygosity) in the nucleus followed the expected pattern of a situation where individuals in generation $t$ are created from individuals in generation $t$–1. Actually, genealogical inbreeding and molecular homozygosity in the nucleus did increase across generations (data not shown). However, at each generation, the commercial population is created from the nucleus and, thus, there is no direct connection between consecutive generations of the commercial population.

The higher or lower level of inbreeding generated by MS depended on the particular relationship among selection candidates. In the first generation, there was a wider range of situations regarding the relationships for MS and, therefore, when decisions were not based on the true values, it had a greater probability of incurring high inbreeding. As the number of generations increased, candidates were more homogeneous and inbreeding was lower than in early generations. Please note that when the true genetic values were used, inbreeding in the commercial population and in the nucleus showed a parallel evolution.

Evolution of ${H}_{o}$ in the nucleus (i.e. under MCM) showed the same trend as that of the genealogical inbreeding. Thus, rates of inbreeding calculated as $\Delta F=\left({F}_{t}-{F}_{t-1}\right)/\left(1-{F}_{t-1}\right)$, were similar regardless of whether they were based on pedigree or molecular information (data not shown).

When the true additive and dominance effects of QTL and the molecular relationships at the QTL positions were used (QTL scenarios), MS yielded higher average phenotypic values than MCM for scenarios with dominance effects (Table 4 and Fig. 1) except for DOM_EQU_10. ${H}_{o}$ (Table 4), and genealogical inbreeding levels (see Additional file 1: Table S1) calculated based on the effects estimated at the non-SNP loci were practically equal under both MS and MCM, but lower than when based on the effects estimated at the SNPs.

**Table 4:** Mean phenotype, observed homozygosity (${H}_{o}$) at the non-SNP loci and the QTL in the commercial population when using the MCM or MS strategy based on the true additive and dominance effect and the genotypes of the QTL under different gene actions

	DOM_VAR		DOM_EQU_10		DOM_EQU_100		DIR_VAR		DOM_EQU_1000	
	MCM	MS	MCM	MS	MCM	MS	MCM	MS	MCM	MS
*t* = 1										
Phenotype	1.14	1.50	0.94	0.74	1.10	1.31	1.13	1.48	1.29	1.5
*H*o non-SNP	0.52	0.53	0.52	0.53	0.52	0.52	0.52	0.52	0.52	0.52
*H*o QTL	0.52	0.53	0.58	0.35	0.53	0.47	0.52	0.51	0.52	0.51
*t* = 4										
Phenotype	3.68	4.00	1.49	1.40	2.88	3.09	3.16	3.54	3.20	3.61
*H*o non-SNP	0.54	0.55	0.54	0.55	0.54	0.54	0.54	0.53	0.54	0.53
*H*o QTL	0.55	0.55	0.99	0.78	0.58	0.52	0.54	0.52	0.53	0.51

Standard errors range from 0.02 to 0.03 for mean phenotype and are always lower than 0.01 for observed homozygosity

*MCM* minimum coancestry mating, *MS* mate selection, *DOM_VAR* additive and dominance effects normally distributed, *DIR_VAR* as in DOM_VAR but with dominance effects all positive, *DOM_EQU_x* all effects equal with *d* = *a*, being × the number of QTL (actually 10, 100 or 1000)

At the QTL, ${H}_{o}$ for traits controlled by 1000 loci were equal with MCM and MS. However, ${H}_{o}$ at the QTL was clearly lower with MS when the trait was determined by fewer loci (see DOM_EQUA_10 or DOM_EQUA_100 in Table 4), which reflects the higher power of controlling simultaneously a small number of loci.

## Discussion

In this study, we investigated, through computer simulations, the possibility of exploiting dominance effects when using genomic tools in the framework of an aquaculture breeding programme, which comprised a nucleus (where selection is performed) and a commercial population (composed of fish destined for market). When the trait under selection exhibited high levels of inbreeding depression, the mate selection strategy (MS), which is explicitly designed to maximize the performance of the offspring in the commercial population, yielded lower average phenotypic values than the alternative strategy of selecting fish based on the EBV and applying subsequently minimum coancestry mating (MCM). However, when inbreeding depression was low or zero (i.e. there was no directional dominance), MS slightly outperformed MCM.

### Estimating dominance

Although several authors have proposed to include dominance in genomic evaluation models to obtain more accurate estimation of breeding values [7–13] or to optimize the mating scheme [6, 14], non-additive genetics effects are usually ignored in animal breeding programs. As mentioned previously, one of the reasons for this is that low accuracies are obtained when dealing with pedigrees that do not allow precise estimation of dominance effects, even when genomic information is available [17]. However, the structure of aquaculture breeding populations, with large full-sib families, facilitates estimation of dominance effects. Our results showed accuracies higher than 50% for estimates of dominance deviations in the scenario with the largest number of SNPs, although there were no relevant increases in accuracy by increasing the number of SNPs above 100,000. It should be noted that, here, family size was 30 and the number of families was 50. Actually, a larger number of offspring per family and, particularly, a larger number of families are common in commercial aquaculture breeding programs and, thus, higher accuracies can be expected. Therefore, the combination of number of families and family sizes required to obtain a significant change in accuracies need to be investigated. In addition, in this study, genomic evaluation was performed using the true variances but these have to be estimated in practice, which would lead to a lower accuracy of estimates of dominance deviations.

### Exploiting dominance

The simulated scenarios mimicked a situation that is common in aquaculture breeding programs, i.e. the fish destined for market come from a commercial population that is created from the breeding nucleus in each generation, such that the production levels required to cope with the demand can be reached. This structure is ideal for the implementation of methodologies that are directed towards exploitation of dominance effects, without interfering with the selection process carried out in the nucleus (which is based on additive effects). Determining the best mating pairs can be done by using MS (to take advantage of dominance effects) or MCM. The latter has proven to be useful in different animal species and particularly in the aquaculture sector (see, for example [34]).

Results from our simulations show that, in most scenarios (i.e. different gene actions for the QTL and different numbers of SNPs genotyped), MCM yield higher or equal phenotypic means in the commercial population than MS. The advantage of MCM may come from its tendency to avoid matings between relatives to a larger extent and, thus, it tends to increase the percentage of heterozygotes across the genome, even at QTL that are not in LD with any SNPs. Although MCM is not directly developed to deal with dominance, the effective control of inbreeding reduces the risk of inbreeding depression. The importance of controlling inbreeding to increase the expected phenotype of the offspring has also been suggested by Sun et al. [35], even if dominance effects are not estimated and used. In contrast, the MS approach promotes heterozygosity exclusively at SNPs for which a significant dominance effect has been estimated. With the simulated parameters, it seems that the accuracy of the estimates of effects is not high enough, leading to suboptimal schemes that generate increased levels of inbreeding (genealogical or molecular), which cause a reduction in performance for the selected trait. Actually, without directional dominance (scenario DOM_VAR) and, therefore, no inbreeding depression, MS outperformed MCM, even when a small number of SNPs was used. Moreover, for the scenario DOM_EQU_10, for which inbreeding depression was around 1.3 SD (per 100% *F*), MS resulted in slightly higher mean phenotypic values in the commercial population at $t$ = 1 when only 100 SNPs were used. For this scenario, differences between MS and MCM were small for subsequent generations and also for other SNPs densities. The connection between the levels of inbreeding (homozygosity) generated and the phenotypic performance in the commercial population is also reflected in the results obtained from additional simulations in which random mating was applied to the selected parents (i.e. no mating allocation) for scenarios DOM_EQU_1000, DOM_EQU_100, DIR_VAR and DOM_VAR using 100,000 SNPs for management of the breeding program. Average phenotypes under random mating always evolved in parallel with phenotypes under MCM but were always lower (see Additional file 2: Figure S1). Differences in phenotypic values between random mating and MCM became smaller as inbreeding depression decreased, and null for the scenario DOM_VAR without inbreeding depression. The average genealogical inbreeding and homozygosity followed a similar trend but were higher under random mating than under MCM (data not shown). This suggests that, compared to random mating, mate allocation (i.e. planned mating) is advantageous and that this superiority relates to the degree of inbreeding/homozygosity generated.

Leroy [36] performed a meta-analysis of published estimates of inbreeding depression for different animal domestic species. These studies were heterogenous in terms of estimation methods and of the estimates obtained. The average inbreeding depression was 0.56 SD at 100% inbreeding, with some estimates as high as five SD (see Figure S1 in [36]). Our results show that, although some advantage can be obtained from the use of MS compared to MCM at the lower bound of the range of published estimates of inbreeding depression, this advantage is small. However, for traits with high levels of inbreeding depression, the disadvantage (i.e. lower phenotype) of using MS versus MCM could be high. Consequently, in general, implementation of MCM instead of MS should be recommended for commercial population.

Dealing with a scenario that included a single population (the selection nucleus), Aliloo et al. [14] compared MCM with a mating design method that tried to maximise the phenotype of the offspring (accounting for dominance) for choosing mating pairs after selection based on EBV. They found significant increases in the average phenotype of the offspring when using estimates of dominance effects compared to using MCM. Notwithstanding, the advantage of MS was only observed when a large weight (actually multiplied by 100) was given to the term related to inbreeding depression (i.e. expected heterozygosity in the offspring) when calculating the expected phenotype of the offspring of each mating pair. The differences with our results may be because, in our implementation of MS, mating pairs were chosen from all selection candidates in the nucleus, with the aim of maximizing the expected phenotype in the offspring, i.e. simultaneously deciding who to select and the mating pairs. It should be noted that this is different from the management scheme presented by [14], in which selection was exclusively on EBV and conducted independently of the mating scheme. Therefore, estimates of dominance effects were only used to determine the mating pairs from the already selected animals.

Toro and Varona [6] also simulated a mate selection process with selection and mating decisions performed in a single step. They found that MS had some advantages (in terms of the average phenotype of the commercial population) over a two-step process with random mating. In our study, comparisons were made with a scenario in which MCM was performed after the selection step, leading to lower levels of inbreeding than in [6] in the reference scenario (i.e. independent selection and mating). Our study shows the importance of the level of inbreeding for the performance of the MS strategy.

In this study, in the scenarios that involve dominance effects, they were simulated independently of the additive effect. However, it has been shown that correlations between additive and dominance effects exist [37, 38]. In such scenarios, inbreeding could be even more important because QTL that have greater additive effects (and, therefore, are under greater selection pressure) tend to be dominant, leading to larger inbreeding depression. The performance of MS in this situation deserves further investigations.

### Effect of SNP density and linkage disequilibrium

It should be noted that, for a given type of gene action, superiority of MCM over MS increased as the number of SNPs used increased (Fig. 2). When moving from 100 to 10,000 SNPs per Morgan, the accuracy of estimates of breeding values and dominance deviations increased (Table 2), leading to higher phenotypic means in the commercial population for both MCM and MS (Table 1). At the same time, the correlation between coancestry computed from SNP genotypes versus QTL genotypes increased with increasing SNP density. Therefore, the control of inbreeding at the QTL with MCM is more efficient with a denser panel of markers (i.e. the percentage of heterozygous individuals is higher), enlarging the advantage of MCM over MS irrespective of the improvements with MS that resulted from more accurate estimation of additive and dominance effects.

Among other factors, the comparison in performance between the MCM and MS strategies also depends on the LD between SNPs and QTL. At $t$ = 0, the LD in the simulated genome was rather low (${r}^{2}$ = 0.09 and 0.02 for distances around 1 and 10 cM, respectively). However, in fish populations, low LD is common. For example, LD was even lower in a coho salmon breeding population [39] (${r}^{2}$ = 0.05 at distance of 0.2 cM). In contrast, Kijas et al. [40] reported higher LD for Tasmanian populations of Atlantic salmon, which is probably due to their lower population sizes in the past. In turbot [41], estimates of LD across the genome of a recently domesticated population were as low as ${r}^{2}$ = 0.10 for SNPs that were 0.005 cM apart. Thus, the parameters simulated in our study are representative of an aquaculture population. In livestock, higher levels of LD are found [42–44] and, therefore, the relative efficiency of MS and MCM will differ from what was observed here.

### Management based on QTL

To better understand the comparison between MS and MCM, we performed simulations by applying these strategies when using the true additive and dominance effects of QTL and their genotypes. In these scenarios, MS outperformed MCM, regardless of the level of inbreeding depression, except in the case of DOM_EQU_10 (see the explanation for this particular outcome below). The superiority of MS over MCM in these simulations gives us some clues on the performance of MS. First, the poor results for MS when using estimates of SNP effects is due to the low accuracy of the estimates and not to the methodology itself. Second, the poor performance of MS based on SNPs is not related to a malfunction (i.e. lack of power to find the best solution) of the optimization algorithm. It should be noted that MCM optimized mating decisions for a 50 $\times$ 50 matrix of selected individuals, while MS dealt with a 750 $\times$ 750 matrix, which represents a much greater computational load and a more complex optimization problem. Notwithstanding, when using the true QTL effects and genotypes, the MS optimization algorithm was able to find better solutions than MCM. In addition, the MS strategy was repeated for some scenarios for which the performance of MS was worse than that of MCM, by using a different optimization method (the simulated annealing algorithm) but the results did not change.

However, for the DOM_EQU_10 scenario, we found that, MCM outperformed MS when using true QTL effects and genotypes, which contrasted with the previous observations. This could be explained in the following way. Let us imagine an extreme case of a trait controlled by a single QTL with complete dominance ($a=d$), and assume that selection candidates are males and females with the *AA* and *Aa* genotypes at the QTL, and that a single pair of individuals is to be selected. Homozygous individuals (*AA*) have a higher breeding value than heterozygous *Aa*, $\left(2\left(1-p\right){\alpha }^{2}\right)$ versus $\left(1-2p\right){\alpha }^{2})$ and, thus, one *AA* male and one *AA* female will be selected. Offspring from the mating of those individuals will all be *AA* and have a genetic (mean phenotypic) value of $a$. When performing MS, decisions are based on the expected genetic value of the offspring of each possible mating pair. Matings *Aa* $\times$ *Aa* will yield lower values, since they generate *aa* individuals, and will never be selected. However, matings *AA* $\times$ *Aa* and *AA* $\times$ *AA* generate offspring with the same mean value because heterozygotes and homozygotes *AA* have the same value ($d=a$). Consequently, with MS, heterozygotes can be selected and, thus, the frequency of the beneficial allele (*A*) will not increase as quickly as it does under MCM. Table 4 shows that the observed homozygosity for QTL becomes much higher with MCM than with MS. The larger the number of QTL, the more complicated it is to find individuals that are homozygous for all or most beneficial alleles, resulting in MS to show its superiority over MCM in our simulations for traits controlled by 100 or 1000 QTL. However, with few QTL (scenario DOM_EQU_10), the situation explained above (i.e. MS selecting more heterozygotes) occurs and MCM outperforms MS. It is important to highlight again that the difference between strategies lies in the values used for decision making: estimated breeding values with MCM against expected offspring values with MS.

### Computational load

The difference in computing time between MCM and MS can be huge and depends on the number of evaluated candidates; but this may not be a problem for its application in a breeding program, since it has to be performed only once per generation. For the scenario DOM_EQUAL_1000 and using 200,000 SNPs, the MS optimization algorithm was 1000 times slower than that of MCM, with 15% of the time spent on the construction of the matrix of expected performances in the offspring. When reducing the number of fish evaluated from each family, these differences in computing time between MS and MCM decreased a lot, with MS being only 50 times slower when 10 candidates per family were considered (65% of the time spent on matrix construction) and 1.7 times when only two individuals per family were evaluated. Note that the MCM optimization always dealt with a 50 $\times$ 50 matrix, while the MS optimization dealt with a 50 $\times$ 50 matrix when evaluating two candidates per family and a 250 $\times$ 250 matrix for 10 candidates per family.

## Conclusions

To create the commercial population, the strategy that involves a two-step process, where fish are selected based on their EBV and then mated following a MCM design, gave higher mean phenotypic values for the commercial population than the strategy that involves a single step through MS in most simulated scenarios. Even for traits with low or no ID, the advantage of MS was never high. Moreover, MCM has the appealing property of reducing inbreeding levels, with a corresponding reduction in inbreeding depression for traits beyond those included in the selection objective. Finally, our results show that controlling inbreeding is very important in the presence of dominance and inbreeding depression. In this sense, another possible strategy would be to implement mate selection but with an explicit restriction on the generation of inbreeding using optimal contributions theory [45]. The computational load that this optimization implies in large populations could be reduced by using differential evolution algorithms [22, 23] or semidefinite programing [46].

## Supplementary Information

**Additional file 1: Table S1.** Mean observed homozygosity (${H}_{o}$) at SNPs, non-SNP loci and QTL and genealogical inbreeding in the commercial population when using the MCM or MS strategy based on the genotypes of a variable number of SNPs (*n*_*SNP*_) under different gene actions.

**Additional file 2: Figure S1.** Mean phenotype in the commercial population across generations when using the MCM and MS strategies or random mating based on genotypes for 100,000 SNPs. (a) scenario DOM_VAR; (b) scenario DOM_EQU_100; (c) scenario DIR_VAR; and (d) scenario DOM_EQU_1000.

## Data Availability

The datasets generated and analysed during the current study are available from the corresponding author on reasonable request.
