# Supplementary material for: Optimum mating designs for exploiting dominance in genomic selection schemes for aquaculture species
Source: Genet Sel Evol. 2021 Feb 10;53:14. doi: 10.1186/s12711-021-00610-9 (PMC7877044; doi:10.1186/s12711-021-00610-9)
Supplement: Supplementary file 1 — Additional file 1: Table S1. Mean observed homozygosity (${H}_{o}$Ho) at SNPs, non-SNP loci and QTL and genealogical inbreeding in the commercial population when using the MCM or MS strategy based on the genotypes of a variable number of SNPs (nSNP) under different gene actions. [file 12711_2021_610_MOESM1_ESM.docx]

**Table S1. Mean observed homozygosity (Ho) in the SNP, the non-SNP loci and the QTLs and the genealogical inbreeding in the commercial population when using the MCM or the MS strategy based on the genotypes of a variable number of SNPs (nSNP) under different gene actions.**

| ***n_SNP_*** |  | **ADD_VAR** | |  | **DOM_VAR** | |  | **DOM_EQU_10** | |  | **DOM_EQU_100** | |  | **DIR_VAR** | |  | **DOM_EQU_1000** | |
| --- | --- | --- | --- | --- | --- | --- | --- | --- | --- | --- | --- | --- | --- | --- | --- | --- | --- | --- |
|  |  | ***MCM*** | ***MS*** |  | ***MCM*** | ***MS*** |  | ***MCM*** | ***MS*** |  | ***MCM*** | ***MS*** |  | ***MCM*** | ***MS*** |  | ***MCM*** | ***MS*** |
| $\boldsymbol{t}$**= 1** |  |  |  |  |  |  |  |  |  |  |  |  |  |  |  |  |  |  |
| 100 | SNP | 0.49 | 0.57 |  | 0.49 | 0.54 |  | 0.49 | 0.54 |  | 0.49 | 0.54 |  | 0.52 | 0.63 |  | 0.49 | 0.53 |
|  | no-SNP | 0.52 | 0.57 |  | 0.52 | 0.54 |  | 0.52 | 0.53 |  | 0.52 | 0.54 |  | 0.52 | 0.63 |  | 0.52 | 0.53 |
|  | QTL | 0.52 | 0.57 |  | 0.52 | 0.54 |  | 0.53 | 0.54 |  | 0.52 | 0.53 |  | 0.52 | 0.61 |  | 0.52 | 0.53 |
|  | Pedigree | 0.00 | 0.09 |  | 0.00 | 0.02 |  | 0.00 | 0.02 |  | 0.00 | 0.02 |  | 0.00 | 0.21 |  | 0.00 | 0.02 |
|  |  |  |  |  |  |  |  |  |  |  |  |  |  |  |  |  |  |  |
| 1000 | SNP | 0.51 | 0.58 |  | 0.51 | 0.56 |  | 0.51 | 0.56 |  | 0.51 | 0.56 |  | 0.51 | 0.55 |  | 0.51 | 0.55 |
|  | no-SNP | 0.52 | 0.58 |  | 0.52 | 0.56 |  | 0.52 | 0.55 |  | 0.52 | 0.56 |  | 0.52 | 0.55 |  | 0.52 | 0.55 |
|  | QTL | 0.52 | 0.58 |  | 0.52 | 0.56 |  | 0.54 | 0.56 |  | 0.52 | 0.55 |  | 0.52 | 0.55 |  | 0.52 | 0.55 |
|  | Pedigree | 0.00 | 0.10 |  | 0.00 | 0.06 |  | 0.00 | 0.06 |  | 0.00 | 0.06 |  | 0.00 | 0.05 |  | 0.00 | 0.05 |
|  |  |  |  |  |  |  |  |  |  |  |  |  |  |  |  |  |  |  |
| 10,000 | SNP | 0.52 | 0.58 |  | 0.52 | 0.62 |  | 0.52 | 0.61 |  | 0.52 | 0.61 |  | 0.52 | 0.60 |  | 0.52 | 0.59 |
|  | no-SNP | 0.52 | 0.58 |  | 0.52 | 0.62 |  | 0.52 | 0.61 |  | 0.52 | 0.61 |  | 0.52 | 0.60 |  | 0.52 | 0.59 |
|  | QTL | 0.52 | 0.58 |  | 0.52 | 0.62 |  | 0.54 | 0.59 |  | 0.52 | 0.60 |  | 0.52 | 0.60 |  | 0.52 | 0.59 |
|  | Pedigree | 0.00 | 0.10 |  | 0.00 | 0.18 |  | 0.00 | 0.17 |  | 0.00 | 0.17 |  | 0.52 | 0.60 |  | 0.00 | 0.14 |
|  |  |  |  |  |  |  |  |  |  |  |  |  |  |  |  |  |  |  |
| 100,000 | SNP | 0.52 | 0.58 |  | 0.52 | 0.63 |  | 0.52 | 0.63 |  | 0.52 | 0.63 |  | 0.52 | 0.62 |  | 0.52 | 0.62 |
|  | no-SNP | 0.52 | 0.58 |  | 0.52 | 0.63 |  | 0.52 | 0.63 |  | 0.52 | 0.63 |  | 0.52 | 0.62 |  | 0.52 | 0.62 |
|  | QTL | 0.52 | 0.58 |  | 0.52 | 0.63 |  | 0.54 | 0.60 |  | 0.52 | 0.61 |  | 0.52 | 0.62 |  | 0.52 | 0.61 |
|  | Pedigree | 0.00 | 0.10 |  | 0.00 | 0.20 |  | 0.00 | 0.21 |  | 0.00 | 0.20 |  | 0.00 | 0.19 |  | 0.00 | 0.19 |
|  |  |  |  |  |  |  |  |  |  |  |  |  |  |  |  |  |  |  |
| 200,000 | SNP | 0.52 | 0.58 |  | 0.52 | 0.63 |  | 0.52 | 0.63 |  | 0.52 | 0.63 |  | 0.52 | 0.62 |  | 0.53 | 0.62 |
|  | no-SNP | 0.52 | 0.58 |  | 0.52 | 0.63 |  | 0.52 | 0.63 |  | 0.52 | 0.63 |  | 0.52 | 0.62 |  | 0.53 | 0.62 |
|  | QTL | 0.52 | 0.58 |  | 0.52 | 0.63 |  | 0.54 | 0.60 |  | 0.52 | 0.61 |  | 0.52 | 0.62 |  | 0.52 | 0.62 |
|  | Pedigree | 0.00 | 0.10 |  | 0.00 | 0.21 |  | 0.00 | 0.21 |  | 0.00 | 0.21 |  | 0.00 | 0.19 |  | 0.00 | 0.19 |
| $\boldsymbol{t}$**= 4** |  |  |  |  |  |  |  |  |  |  |  |  |  |  |  |  |  |  |
| 100 | SNP | 0.56 | 0.58 |  | 0.56 | 0.61 |  | 0.56 | 0.60 |  | 0.55 | 0.59 |  | 0.55 | 0.59 |  | 0.55 | 0.59 |
|  | no-SNP | 0.55 | 0.57 |  | 0.56 | 0.57 |  | 0.55 | 0.56 |  | 0.55 | 0.56 |  | 0.55 | 0.56 |  | 0.55 | 0.56 |
|  | QTL | 0.56 | 0.57 |  | 0.56 | 0.57 |  | 0.62 | 0.63 |  | 0.55 | 0.56 |  | 0.55 | 0.56 |  | 0.54 | 0.55 |
|  | Pedigree | 0.06 | 0.08 |  | 0.06 | 0.08 |  | 0.05 | 0.07 |  | 0.05 | 0.07 |  | 0.06 | 0.08 |  | 0.04 | 0.06 |
|  |  |  |  |  |  |  |  |  |  |  |  |  |  |  |  |  |  |  |
| 1000 | SNP | 0.54 | 0.59 |  | 0.54 | 0.57 |  | 0.54 | 0.56 |  | 0.54 | 0.56 |  | 0.54 | 0.56 |  | 0.54 | 0.56 |
|  | no-SNP | 0.55 | 0.59 |  | 0.55 | 0.57 |  | 0.54 | 0.55 |  | 0.54 | 0.56 |  | 0.54 | 0.56 |  | 0.54 | 0.56 |
|  | QTL | 0.55 | 0.59 |  | 0.55 | 0.57 |  | 0.71 | 0.71 |  | 0.55 | 0.57 |  | 0.54 | 0.56 |  | 0.54 | 0.55 |
|  | Pedigree | 0.05 | 0.12 |  | 0.05 | 0.08 |  | 0.04 | 0.05 |  | 0.05 | 0.07 |  | 0.04 | 0.06 |  | 0.04 | 0.06 |
|  |  |  |  |  |  |  |  |  |  |  |  |  |  |  |  |  |  |  |
| 10,000 | SNP | 0.54 | 0.60 |  | 0.54 | 0.58 |  | 0.54 | 0.56 |  | 0.54 | 0.57 |  | 0.54 | 0.56 |  | 0.54 | 0.56 |
|  | no-SNP | 0.55 | 0.61 |  | 0.54 | 0.58 |  | 0.54 | 0.56 |  | 0.54 | 0.57 |  | 0.54 | 0.56 |  | 0.54 | 0.56 |
|  | QTL | 0.55 | 0.61 |  | 0.55 | 0.58 |  | 0.74 | 0.74 |  | 0.56 | 0.57 |  | 0.54 | 0.56 |  | 0.54 | 0.56 |
|  | Pedigree | 0.05 | 0.15 |  | 0.05 | 0.10 |  | 0.04 | 0.06 |  | 0.05 | 0.08 |  | 0.04 | 0.08 |  | 0.05 | 0.07 |
|  |  |  |  |  |  |  |  |  |  |  |  |  |  |  |  |  |  |  |
| 100,000 | SNP | 0.54 | 0.60 |  | 0.54 | 0.59 |  | 0.54 | 0.56 |  | 0.54 | 0.58 |  | 0.54 | 0.57 |  | 0.54 | 0.57 |
|  | no-SNP | 0.54 | 0.60 |  | 0.54 | 0.59 |  | 0.54 | 0.56 |  | 0.54 | 0.58 |  | 0.54 | 0.57 |  | 0.54 | 0.57 |
|  | QTL | 0.55 | 0.60 |  | 0.55 | 0.59 |  | 0.74 | 0.74 |  | 0.56 | 0.58 |  | 0.54 | 0.56 |  | 0.54 | 0.56 |
|  | Pedigree | 0.05 | 0.15 |  | 0.05 | 0.13 |  | 0.04 | 0.08 |  | 0.04 | 0.10 |  | 0.04 | 0.08 |  | 0.04 | 0.08 |
|  |  |  |  |  |  |  |  |  |  |  |  |  |  |  |  |  |  |  |
| 200,000 | SNP | 0.55 | 0.60 |  | 0.55 | 0.60 |  | 0.54 | 0.57 |  | 0.54 | 0.58 |  | 0.54 | 0.57 |  | 0.55 | 0.57 |
|  | no-SNP | 0.55 | 0.60 |  | 0.55 | 0.59 |  | 0.54 | 0.57 |  | 0.54 | 0.58 |  | 0.54 | 0.57 |  | 0.55 | 0.57 |
|  | QTL | 0.55 | 0.60 |  | 0.55 | 0.59 |  | 0.75 | 0.75 |  | 0.56 | 0.58 |  | 0.54 | 0.56 |  | 0.54 | 0.56 |
|  | Pedigree | 0.05 | 0.14 |  | 0.05 | 0.13 |  | 0.04 | 0.08 |  | 0.05 | 0.10 |  | 0.04 | 0.09 |  | 0.04 | 0.08 |

*MCM*: Minimum coancestry mating; *MS*: Mate selection; DOM_VAR: additive and dominance effects normally distributed; DIR_VAR: as in DOM_VAR but with dominance effects all positive; DOM_EQU_x: all effects equal with *d* = *a*, being x the number of QTLs (actually 10, 100 or 1,000).
