# Supplementary material for: Optimum mating designs for exploiting dominance in genomic selection schemes for aquaculture species
Source: Genet Sel Evol. 2021 Feb 10;53:14. doi: 10.1186/s12711-021-00610-9 (PMC7877044; doi:10.1186/s12711-021-00610-9)
Supplement: Supplementary file 2 — Additional file 2: Figure S1. Mean phenotype in the commercial population across generations when using the MCM and MS strategies or random mating based on genotypes for 100,000 SNPs. (a) scenario DOM_VAR; (b) scenario DOM_EQU_100; (c) scenario DIR_VAR; and (d) scenario DOM_EQU_1000. [file 12711_2021_610_MOESM2_ESM.docx]

**c)**

**d)**

**b)**

**a)**
